# Tolerability of intranasal esketamine, a case series of 15 patients

**DOI:** 10.1192/j.eurpsy.2024.1128

**Published:** 2024-08-27

**Authors:** I. Álvarez Correa, C. Delgado Marmisa, H. J. Vizcaíno Herrezuelo, L. Egüen Recuero, E. Garrido Dobrito, L. D. Téllez de Cepeda, L. Gayubo Moreo, B. Sanz-Aránguez Ávila, L. Caballero Martínez, R. de Arce Cordón

**Affiliations:** ^1^ M.I.R. Psiquiatría; ^2^ Enfermería Psiquiatría; ^3^ F.E.A Farmacología Clínica; ^4^ F.E.A Psiquiatría; ^5^ Jefe de Sección Psiquiatría; ^6^Jefa de Servicio Psiquiatría, Hospital Universitario Puerta de Hierro Majadahonda, Madrid, Spain

## Abstract

**Introduction:**

Intranasal esketamine has recently been approved for the treatment of treatment-resistant depression in adults, with different studies showing its efficacy and tolerability. However, the real-world tolerability of this treatment is still unclear.

**Objectives:**

Evaluate the tolerability of intranasal esketamine in a case series of 15 patients.

**Methods:**

Our case series includes 15 patients, who received treatment with intranasal esketamine during 2022-2023. In order to evaluate the tolerability of intranasal esketamine, patients were asked to complete the TSQM and a side effect questionnaire on different moments of the treatment (one week, six weeks and six months after the beginning of the treatment).

**Results:**

The most common adverse effects were dissociation, dizziness, and somnolence, which resolved within the hours following the administration. All of them were mild or moderate in severity, having a minor impact on the patient, so none of the patients discontinued the treatment due to adverse effects. Other adverse effects noticed were: transitory increment of blood pressure in several patients, and worsening of obsessions in a patient with previous obsessive-compulsive symptoms.

**Conclusions:**

Our data suggests that intranasal esketamine is well tolerated, with transient and mild adverse effects. In all cases the risk-benefit ratio must be evaluated, but until more studies are done, it seems to be a safe treatment for treatment-resistant depression.

**Disclosure of Interest:**

None Declared

